# Diagnostic and therapeutic challenges in acute retinal necrosis; an update

**DOI:** 10.1038/s41433-024-03028-x

**Published:** 2024-03-22

**Authors:** Dimitrios Kalogeropoulos, Farid Afshar, Chris Kalogeropoulos, Georgios Vartholomatos, Andrew John Lotery

**Affiliations:** 1 Southampton Eye Unit, University Hospital Southampton, Southampton, UK; 2 Department of Ophthalmology, Faculty of Medicine, School of Health Sciences, University of Ioannina, Ioannina, Greece; 3 Hematology Laboratory, Unit of Molecular Biology, University Hospital of Ioannina, Ioannina, Greece; 4 Faculty of Medicine, University of Southampton, Southampton, UK

**Keywords:** Uveal diseases, Viral infection

## Abstract

Acute retinal necrosis (ARN) is a rare but severe ophthalmic pathology defined by panuveitis, retinal necrosis, and high rates of retinal detachment. ARN may lead to poor visual outcomes even if promptly diagnosed and treated. ARN may present with a wide spectrum of clinical findings compatible with panuveitis including anterior uveitis, scleritis, vitritis, necrotizing retinitis, occlusive vasculitis, and optic disc edema. The American Uveitis Society introduced clinical criteria in 1994 for the diagnosis of ARN, while more recent criteria have been proposed by the Standardization of Uveitis Nomenclature (SUN) Working Group and the Japanese ARN Study Group. Multimodal imaging is a valuable tool in evaluating patients with ARN, particularly in unusual cases, while utilizing retinal imaging and applying AI algorithms in these areas of clinical research could be highly beneficial. Over the last few years, significant progress has been made in achieving timely diagnosis and treatment. The precise identification of the viral cause in suspected ARN cases has been greatly enhanced by the advancements in PCR techniques and flow cytometry used for intraocular fluids. systemic (intravenous or oral) antivirals with adjunctive intravitreal antiviral therapy are recommended as first-line therapy to reduce disease severity, the risk of vision loss, and retinal detachment incidence. Although aciclovir was the first existing antiviral agent, at present many clinicians prefer high-dose valaciclovir orally or intravenous aciclovir combined with intravitreal foscarnet. Despite significant progress in diagnosing and treating ARN, further research is needed to improve visual outcomes in this challenging clinical condition.

## Abstract (zh)

急性视网膜坏死 (ARN) 是一种临床少见但发病严重的以全葡萄膜炎、视网膜坏死和视网膜脱离为特征的眼科疾病。即使诊断和治疗及时, ARN仍可导致视力不佳。ARN可能表现与全葡萄膜炎相关的多种临床表现, 包括前葡萄膜炎、巩膜炎、玻璃体炎、坏死性视网膜炎、闭塞性视网膜血管炎和视盘水肿。美国葡萄膜炎学会于1994年引入ARN诊断的临床标准, 而最近的诊断标准由葡萄膜炎命名标准化工作组 (SUN) 和日本ARN研究组提出。多模态成像是评估ARN的有价值工具, 尤其在少见病例中, 利用视网膜成像并在临床研究中应用人工智能算法可能有益。在过去几年中, ARN在及时诊断和治疗方面取得了重要进展。PCR技术和用于眼内液的流式细胞学检测的进步大大提高了疑似ARN病例病毒的精准检测。系统性 (静脉或口服) 抗病毒药物与玻璃体内抗病毒药物推荐作为首选治疗, 以降低疾病严重程度、视力丧失的风险和视网膜脱离的发生率。尽管阿昔洛韦是首选抗病毒药物, 但目前许多临床医生更喜欢大剂量的伐昔洛韦口服, 或静脉注射阿昔洛韦联合玻璃体内膦甲酸注射。ARN尽管在诊断和治疗方面取得了重大进展, 但需要进一步的研究来提高这一棘手疾病的视力结局。

## Introduction

Acute retinal necrosis (ARN) is a rare but severe ophthalmic pathology defined by panuveitis, retinal necrosis, and high rates of retinal detachment [1]. ARN may lead to poor visual outcomes even if promptly diagnosed and treated [2, 3]. The primary goal of treating ARN with intravenous antiviral drugs such as aciclovir, valaciclovir, and ganciclovir is to halt the progression of the disease in the affected eye and prevent it from developing to the other eye [4, 5]. The use of polymerase chain reaction analysis of aqueous or vitreous fluid can aid in the prompt diagnosis of ARN [2]. Additional treatment options may include intravitreal antiviral agents, as well as topical and oral corticosteroids, antithrombotic treatment, prophylactic laser barricade, and vitrectomy, but the effectiveness of these treatments can vary [2]. A combination of therapies may help to reduce the risk of severe vision loss and improve visual acuity, but more research is needed in this area. The focus of this review is to highlight the latest developments in the diagnosis and treatment of ARN. Specifically, it explores the use of a combination of antiviral therapy and surgical interventions to manage the disease.

### Historical aspects

In 1971, a Japanese ophthalmologist named Akira Urayama [6] first identified ARN as a unilateral disease, characterized by panuveitis and retinal arteritis that ultimately led to retinal detachment and necrotizing retinitis. A few years later, the term “BARN” was coined by Young and Bird [7] to describe the occurrence of bilateral ARN (BARN). The definitive identification of a viral cause affecting all layers of the retina was shown by Culbertson et al. [8] using electron microscopy. The examination of tissue samples revealed severe acute necrosis of the retina, retinal arteritis, and the presence of eosinophilic intranuclear inclusions in retinal cells. It is now widely accepted that the primary cause of ARN is the varicella-zoster virus (VZV), with herpes simplex virus 1 and 2 (HSV-1 and 2) being the second most common cause, and cytomegalovirus (CMV) being a less common cause [9]. Although the Epstein-Barr virus (EBV) has been occasionally linked to ARN [10], it is generally not considered pathogenic in most cases [11].

### Epidemiology

ARN is still considered a rare yet dreadful clinical entity. In the United Kingdom, two studies were conducted by the British Ophthalmological Surveillance Unit (BOSU) utilizing a monthly surveillance system [12, 13] and reported an incidence rate ranging from 0.5 to 0.63 cases per million population per year [13]. In Japan, ARN accounts for around 1.3-1.4% of all uveitis cases [14, 15]. There is no known gender or race predilection for ARN; however, a genetic link has been found in Caucasians with the HLA-DQw7 antigen and HLA-Bw62 phenotype, suggesting a potential immune predisposition to developing ARN [16]. Older individuals are more likely to develop ARN due to the varicella-zoster virus (HZV) and herpes simplex virus 1 (HSV-1), whereas those under 25 years old are more frequently affected by HSV-2 [9, 17]. It has been reported that HSV-2 can cause ARN even up to 30 years after a neonatal infection [18]. ARN typically affects immunocompetent and healthy adults, in contrast to Progressive Outer Retinal Necrosis (PORN), another type of herpetic retinopathy that occurs in immunocompromised individuals [2]. Interestingly, one report showed a seasonal variation in ARN incidence, with the highest rates occurring during winter and spring [19]. ARN can occur several years after the primary infection or follow a systemic herpetic infection (e.g., herpetic dermatitis or encephalitis) [20]. Interestingly, studies have documented a history of a prior herpetic infection in up to 55% of ARN patients [12, 21]. It is important to consider ARN in patients with herpes encephalitis, as the incidence of ARN in these patients has been reported to be 4% to 8% [22].

### Clinical features and complications

**Fig. 1:**
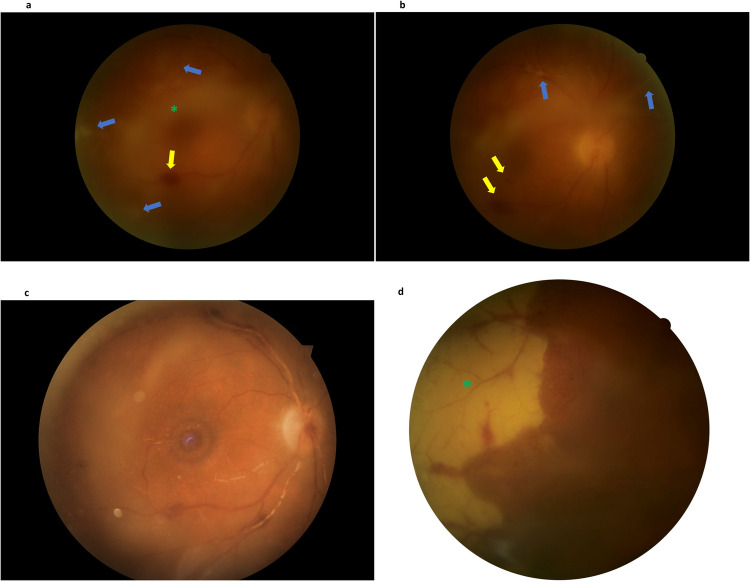
A 63-year-old gentleman with a free ophthalmic and systemic history presented to the eye casualty with gradual worsening of his right eye vision over the last 48 h. At presentation, the visual acuity of the affected eye did not exceed counting fingers at a 1-metre distance. **a** Dilated fundoscopy revealed dense vitritis (vitreous haze: 3 + ) (green asterisk) with distinct, multifocal, peripheral, confluent patches of yellowish infiltrates in the deep retina (blue arrows). Retinal haemorrhages can also be observed (yellow arrow). Aqueous humour was obtained with anterior chamber paracentesis and was sent for polymerase chain reaction (PCR) and flow cytometry. Due to the high suspicion of acute retinal necrosis patient was empirically started on antiviral treatment. PCR was positive for varicella-zoster virus. **b** Signs of occlusive vasculitis (blue arrows) with limited retinal haemorrhages (yellow arrows). **c** Patient was initially treated with intravenous aciclovir 750 mg three times daily. On the 6th day of this course 16 mg of oral prednisolone was added to the therapeutic regimen. Due to the occlusive vasculitis oral aspirin 100 mg once daily was also administered to the patient. He received an overall of 11 intravitreal ganciclovir injections Fundoscopic image after two months. Best corrected Snellen visual acuity remained stable at 5/10. **d** Examination of the peripheral retina with evidence of peripheral retinal atrophy.

ARN may present with a wide spectrum of clinical findings compatible with panuveitis including anterior uveitis, scleritis, vitritis, necrotizing retinitis, occlusive vasculitis, and optic disc oedema [2, 3]. Initially, ARN may manifest with mild and nonspecific symptoms such as redness, photophobia, floaters, blurry vision, and pain. Most cases are confined to a single eye, but some cases may involve both eyes (BARN). The clinical features of ARN can be divided into two phases. In the first phase, anterior chamber reaction and typically granulomatous keratic precipitates can be observed. A dilated fundoscopy may reveal varying degrees of vitritis with distinct, multifocal, confluent patches of yellowish-white infiltrates in the deep retina and the retinal pigment epithelium (RPE) [Fig. 1a, b]. These infiltrates typically begin in the peripheral regions of the eye, and there may be signs of vasculitis (usually arteritis) with limited retinal haemorrhages. Atypical features of ARN, such as Kyrieleis arteriolitis [23], segmental granular lesions aligned along the retinal vessels [24], and choroidal involvement [25] have also been reported. As the disease progresses, necrosis occurs, vitreous inflammation increases and the peripheral lesions rapidly spread toward the posterior pole. In the late cicatricial phase, retinal atrophy [Fig. 1c, d] and vitreous traction develop due to inflammatory changes, often leading to the development of a retinal detachment (RD) [11–13, 26]. Despite antiviral treatment, the incidence of RD in ARN cases can vary from 20% to 60% [1]. According to a recent systematic review and meta-analysis, approximately 2% of eyes with ARN have a RD at presentation, while 47% of cases eventually develop a RD over the course of the disease [27].

It is expected that a greater extent of retinal changes in ARN patients can result in a higher risk of RD and a worse visual prognosis [1, 27, 28]. However, there is currently no uniform system to classify the extent of retinal changes in ARN. Some proposed classification systems include the cytomegalovirus retinitis classification system [29] and others based on the number of quadrants [27, 30] or the percentage of retina involved [31]. These classifications can be difficult to implement due to the vitreous inflammation and haze characteristic of ARN. Other factors that may worsen visual prognosis in ARN include VZV as the causative virus [27], more posterior location of retinitis [32], longer duration of symptoms before diagnosis [20], worse presenting visual acuity [12, 30, 32], and optic nerve involvement. ARN may also lead to other complications such as ocular hypotony, macular oedema, proliferative vitreoretinopathy (PVR), epiretinal membrane (ERM), optic atrophy, and phthisis.

### Diagnostic approach

#### Diagnostic criteria

**Fig. 2:**
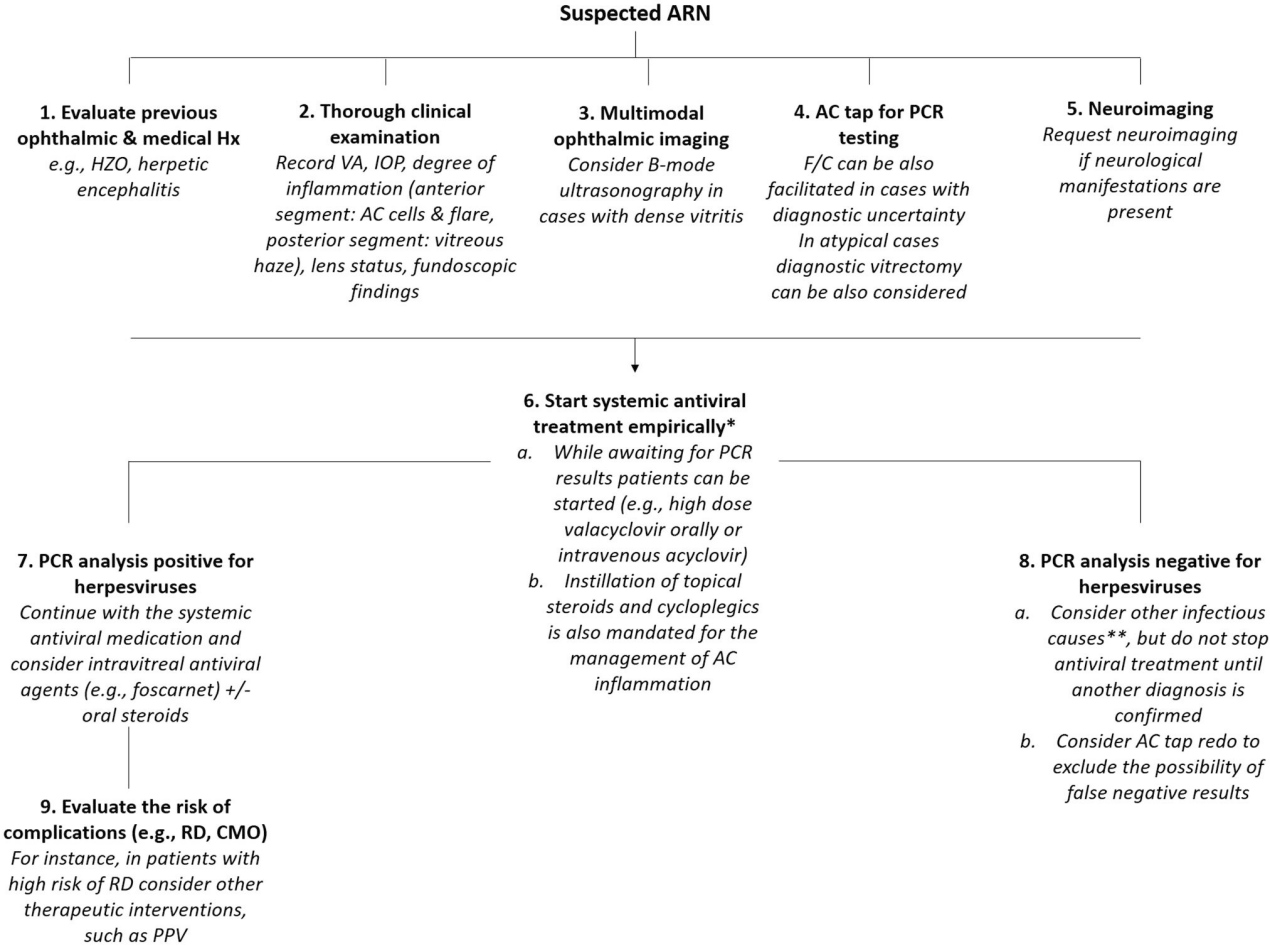
Suggested diagnostic and therapeutic algorithm for the management of patients with suspected acute retinal necrosis. ARN is a rare but severe ophthalmic pathology defined by panuveitis, retinal necrosis, and high rates of retinal detachment. ARN may lead to poor visual outcomes even if promptly diagnosed and treated. The primary goal of treating ARN is to halt the progression of the disease in the affected eye and prevent it from developing to the other eye. Clinicians must always inquire about a possible history of a prior herpetic infection, and request medical assessment from other specialties (e.g., neurology) and neuro-imaging (e.g., in cases with herpes encephalitis) when necessary. The precise identification of the viral cause in suspected ARN cases has been greatly enhanced by the advancements in PCR techniques used for intraocular fluids. AC tap can be obtained and used for PCR testing and flow cytometric analysis to establish a diagnosis of ARN. Systemic treatment can be commenced empirically while waiting for the laboratory results. Further treatments (e.g., intravitreal antiviral agents or oral steroids) can be also added to the therapeutic regimen. Multimodal imaging is a valuable tool in evaluating patients with ARN, particularly in unusual cases. Close follow-up is mandatory to control the inflammatory process and evaluate the risk of potential complications (e.g., CMO, RD). AC anterior chamber, ARN acute retinal necrosis, CMO cystoid macular oedema, HZO herpes zoster ophthalmicus, Hx history, IOP intraocular pressure, PCR polymerase chain reaction, PPV pars plana vitrectomy, RD retinal detachment, VA visual acuity.

The American Uveitis Society introduced clinical criteria in 1994 for the diagnosis of ARN [33], while more recent criteria have been proposed by the Standardization of Uveitis Nomenclature (SUN) Working Group [34] and the Japanese ARN Study Group [35]. The aforementioned criteria are summarized in Table 1. Furthermore, our suggested diagnostic and therapeutic algorithm for the management of patients with ARN is outlined in Fig. 2.

**Table 1:** Available diagnostic criteria for ARN.

The Executive Committee of the American Uveitis Society (1994)	The Japanese ARN Study Group (2015)	The Standardization of Uveitis Nomenclature (SUN) Working Group (2021)
1. One or more foci of retinal necrosis with discrete borders located in the peripheral retina 2. Rapid progression in the absence of antiviral therapy 3. Circumferential spread 4. Evidence of occlusive vasculopathy with arterial involvement 5. A prominent inflammatory reaction in the vitreous and anterior chamber	1. Ocular findings in the early stage 1a. Anterior chamber cells or mutton-fat keratic precipitates 1b. Yellow-white lesion(s) in the peripheral retina (granular or patchy in the early stage, then gradually merging) 1c. Retinal arteritis 1d. Hyperemia of the optic disc 1e. Inflammatory vitreous opacities 1f. Elevated intraocular pressure 2. Clinical courses 2a. Rapid expansion of retinal lesion(s) circumferentially 2b. Development of retinal break or retinal detachment 2c. Retinal vascular occlusion 2d. Optic atrophy 2e. Response to antiviral agents 3. Virologic testing of intraocular fluids Positive by either PCR or Goldmann–Witmer coefficient for HSV-1, HSV-2, or VZV Classification: 1. Virus-confirmed ARN Presence of ocular findings 1a and 1b, the presence of any 1 of the 5 clinical courses, and a positive virologic test result 2. Virus-unconfirmed ARN Presence of 4 of the 6 ocular findings including 1a and 1b, the presence of any 2 of the 5 clinical courses, and a negative virologic test result or when virologic testing has not been performed	1. Necrotizing retinitis involving the peripheral retina AND (either #2 OR #3) 2. Evidence of infection with HSV or VZV a. Positive PCR^a^ for either HSV or VZV from either an aqueous or vitreous specimen 3. Characteristic clinical picture a. Circumferential or confluent retinitis AND b. Retinal vascular sheathing and/or occlusion AND c. More than minimal vitritis^a^ Exclusions 1. Positive serology for syphilis using a treponemal test 2. Intraocular specimen PCR-positive for cytomegalovirus or Toxoplasma gondii (unless there is an immune compromise, morphologic evidence for >1 infection, the characteristic clinical picture of acute retinal necrosis, and the intraocular fluid specimen has a positive PCR for either HSV or VZV)

*ARN* acute retinal necrosis, *HSV* herpes simplex virus, *PCR* polymerase chain reaction, *VZV* varicella-zoster virus.

^a^Vitritis criterion not required in immunocompromised patients.

#### The role of multimodal imaging

Multimodal imaging is a valuable tool in evaluating patients with ARN, particularly in unusual cases [36]. Fundus photography is essential in all cases, playing a crucial role in both diagnosis and ongoing monitoring [3, 20]. The significance of ultra-widefield imaging has been underscored due to its ability to offer a broader view of the posterior segment, thereby exposing additional peripheral lesions. This imaging technique could prove especially beneficial in several pathologies, including ARN. Since a considerable portion of their manifestations is expected to be situated in the mid-periphery and distal periphery, ultra-widefield imaging becomes particularly valuable in these cases [3, 20]. Fluorescein angiography can be useful in providing additional details that may not be visible during the fundoscopic examination, but its usefulness can be limited due to vitritis. Although it is not a diagnostic tool, it can reveal signs of occlusive arteritis and areas of capillary nonperfusion. The choroidal vasculature is typically affected, and areas of early hypofluorescence and late staining consistent with ischaemia-induced inflammatory changes may be visible. Diffuse leakage from retinal vessels due to active vasculitis may be seen as intense extravasation of dye. Early optic nerve involvement is common, and hyperfluorescence of the optic nerve can be observed on angiography [37]. The use of B-scan ultrasonography can be beneficial in detecting the onset of retinal detachment, particularly when limited visibility is present due to vitritis. Ultrasonography has the capability of penetrating through the haze of vitritis and identifying the elevation of the optic nerve head, along with the expansion of the optic nerve sheath [37]. Ultra-wide-field fundus imaging is especially useful in detecting and recording retinal lesions in ARN, especially those located at the periphery or concealed behind opaque media. This imaging method can provide critical information about the patient’s visual prognosis [38]. Ward and Reddy [39] observed that fundus autofluorescence (FAF) imaging can be used to identify and describe pathological changes in the neurosensory retina and RPE that occur in ARN. High contrast autofluorescence patterns can indicate disease activity borders in ARN, which can aid in monitoring disease progression. Optical coherence tomography (OCT) can also provide essential information in cases with early macular involvement. According to Jain et al. [40], hyperreflectivity and thickening of the inner plexiform layer were the initial changes seen on spectral domain OCT (SDOCT), followed by the involvement of all retinal layers corresponding to the yellowish-white lesion. The hyperreflectivity seen on SDOCT correlates with histopathologic evidence of oedema in the inner retinal layers, caused by occlusive vasculopathy of the arteries. En-face widefield OCT angiography (OCTA) can be utilized to non-invasively monitor changes in retinal vessel architecture in ARN over time [41]. However, OCTA artifacts caused by intraocular inflammation make interpretation difficult and will likely continue to be problematic in the future. Therefore, replacing fluorescein angiography completely may be challenging for some time due to issues with image clarity.

#### Diagnostic modalities

##### Polymerase Chain Reaction (PCR)

The precise identification of the viral cause in suspected ARN cases has been greatly enhanced by the advancements in PCR techniques used for intraocular fluids. This has resulted in a considerably high rate of virus detection, ranging from 79–100%, as reported in [42]. Due to the high sensitivity of PCR testing, if the results are negative, the ophthalmologist should either obtain another sample or explore other possible causes of inflammation. It has been observed [43] that there are no significant differences in the detection rates of aqueous and vitreous fluid samples. Aqueous humour is generally preferred as anterior chamber paracentesis is safer and less invasive compared to vitreous biopsy [44]. Studies have reported varying levels of sensitivity for herpesvirus PCR in aqueous humour samples, ranging from 84% to 100% [9]. Similar tests performed on vitreous samples have yielded values between 77.9% and 100% [26]. In addition to detecting the virus, quantitative PCR can also be useful in monitoring the levels of intraocular DNA in patients undergoing treatment or experiencing refractory cases to systemic and intravitreal treatment. By tracking the viral load, useful information can be obtained about treatment resistance and prognosis [1].

##### Goldmann–Witmer Coefficient (GWC)

Initially, serum antibody titres were investigated to assist in diagnosing ARN. However, their interpretation proved challenging due to the high prevalence of antibodies to the viruses that cause ARN in most adults [45]. Additionally, serum antibody levels may not be elevated despite the significant reactivation of the virus in the eye in cases of ARN [46]. While serum antibodies alone are not useful for diagnosing ARN, a comparison of intraocular to serum antibodies can be used to calculate the Goldmann–Witmer coefficient (GWC), which has a high diagnostic value [45, 47]. The GWC has been proposed as a complementary diagnostic tool to PCR analysis of intraocular fluids for infectious uveitis. A GWC of 6 or higher is considered positive for intraocular infection, while a titre between 1 and 5 is considered suspect and a titre below 1 is negative [46]. However, this method has its limitations, including inadequate intraocular antibody production in the early onset of the disease and the variation in the positivity of the GWC over time from the onset of ARN [48]. Therefore, obtaining PCR of aqueous humour is typically recommended as the first-line test in suspected cases of ARN, with GWC calculation considered only if diagnostic challenges persist [47].

##### Flow cytometry (FC)

**Fig. 3:**
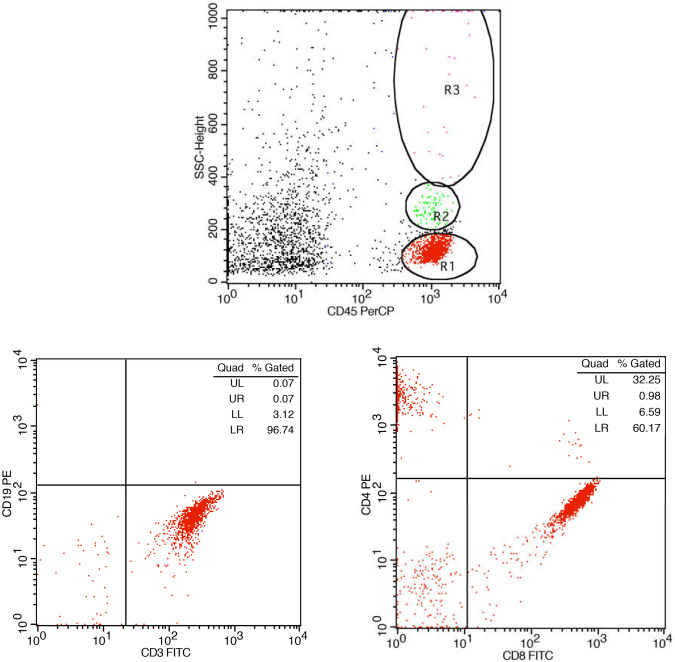
Flow cytometry analysis of aqueous humour. CD45 marker and side scatter are used to characterize lymphocytes (gated at region R1). In gated lymphocytes, further characterization of lymphocyte sub-populations is made by using CD3/CD19 (T lymphocytes /B lymphocytes) and CD4/CD8 (T cell sub-populations). Percentages of cell populations are presented in the upper right of each quadrant.

In addition to PCR testing of intraocular fluids, flow cytometric (FC) analysis can be utilized to investigate lymphocyte subsets in the aqueous or vitreous humour [Fig. 3] and peripheral blood [49]. Kang et al. [50] reported that patients with VZV-induced ARN exhibit unique T lymphocyte subsets and cytokine profiles in intraocular fluids compared to those with non-infectious ARN. A high proportion of CD8 + T lymphocytes and low CD4/CD8 T cell ratios could potentially be used as a biomarker for diagnosing viral-infectious uveitis. Studying T lymphocytes at the site of inflammation could serve as a valuable research tool for distinguishing between viral and non-viral uveitis.

#### Differential diagnosis

Diagnosing ARN can be difficult as many infectious and non-infectious conditions present similar symptoms and clinical features, such as PORN, CMV retinitis, toxoplasmic retinochoroiditis, syphilis, lupus vasculitis, Behçet’s disease, sarcoidosis [2, 3], and bacterial or fungal chorioretinitis [44]. Additionally, primary vitreoretinal lymphoma or leukaemia may show clinical signs that resemble ARN [51].

**Fig. 4:**
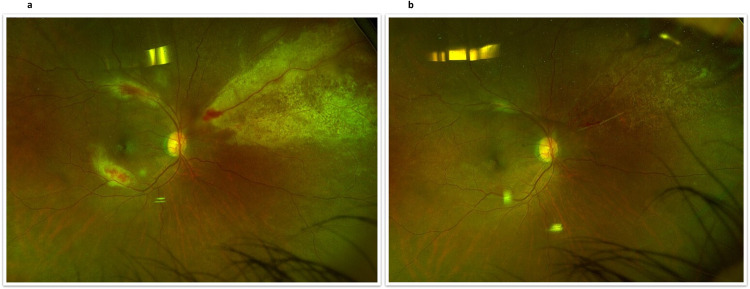
CMV retinitis. Fundoscopic view (right eye) of a patient with CMV retinitis before (**a**) and after (**b**) treatment.

PORN and CMV retinitis are typically seen in individuals with severely compromised immune system (e.g., those diagnosed with AIDS). CMV retinitis (Fig. 4a, b) is, in fact, the primary cause of blindness among individuals diagnosed with AIDS [52]. In contrast with ARN, it is believed that PORN is almost exclusively attributed to the VZV [53].

### Further diagnostic considerations

#### Neuroimaging

Association with viral meningoencephalitis has been reported in ARN cases [20]. These patients may present with a plethora of clinical features ranging from meningitis and meningoencephalitis to uveitis, which constitute the so-called uveomeningeal syndrome [54]. In that case, further investigation may be necessary, and a lumbar puncture may be warranted. Computed tomography (CT) of the orbits can confirm the optic nerve sheath enlargement and associated optic nerve oedema [50]. In select cases, magnetic resonance imaging (MRI) can help to reveal lesions of the optic tract, chiasm, and the lateral geniculate body, which can occur due to axonal spread [55].

#### Laboratory and Serum Testing

Before starting antiviral therapy, it is recommended to conduct laboratory testing, which should comprise a baseline complete blood count, liver function panel, and tests of renal function to keep track of drug toxicity and subsequent dosage adjustments. This is especially crucial in patients with renal failure, including end-stage renal disease and those on dialysis. It must be underlined though that upon making a clinical diagnosis of ARN, treatment should commence promptly, without waiting for laboratory confirmation [3]. Other infectious causes, such as tuberculosis, toxoplasmosis, syphilis, and human immunodeficiency virus, can be ruled out via laboratory testing [44]. However, serum testing for herpesvirus antibodies is not recommended and does not contribute to the diagnosis of ARN.

### Therapeutic approach

Clinical management of the potentially devastating panuveitis syndrome of ARN has mainly relied on retrospective case series, case reports, and expert opinions. However, significant progress has been made in achieving timely diagnosis and treatment. For cases related to HSV or VZV, it is common for the infection to be a result of reactivation from the latent state following previous infections. Herpes viruses can remain dormant in cranial ganglia and migrate in the retina through axons. It is widely recognized that antiviral treatment does not eliminate the virus but rather maintains a balance between the host’s immune response and the virus’s potential to reproduce [20]. Over time, therapeutic strategies for ARN have varied, including differences in the timing of intervention and surgical approaches. Currently, there are six antiviral drugs commercially available namely aciclovir, ganciclovir, valaciclovir, valganciclovir, and foscarnet. This section briefly discusses the route of administration and mechanism of action. It is worth mentioning that although aciclovir was the first existing antiviral agent, at present many clinicians prefer high-dose valaciclovir orally or intravenous aciclovir combined with intravitreal foscarnet [3].

#### Systemic antiviral treatment

Systemic antiviral agents have been shown to improve outcomes, such as decreased optic nerve involvement and regression of retinal lesions, as well as reduced involvement of the fellow eye [4, 5]. Intravenous administration of aciclovir was initially the only available therapeutic option. Aciclovir is a guanine analogue that necessitates a virus-specific thymidine kinase for activation and specifically obstructs the viral DNA polymerase. As a result, it targets solely those cells which are infected with HSV-1, HSV-2, and VZV, and exhibits minimum toxicity towards other cells [56].

Recent research suggests that novel oral antivirals with improved bioavailability, as opposed to oral aciclovir, can be considered for initial therapy, allowing for outpatient management. Valaciclovir is an orally administered prodrug that undergoes first-pass intestinal and/or hepatic metabolism to convert into aciclovir. While intravenous aciclovir reaches its maximum concentration faster, oral valaciclovir can achieve inhibitory vitreous levels and is comparable in terms of efficacy in terms of time to regression of retinitis, final VA, and RD [57]. Furthermore, oral treatment may be a cost-effective alternative to inpatient intravenous therapy [42]. Both aciclovir and valaciclovir are associated with common side effects such as rash, headache, and gastrointestinal symptoms. Additionally, close monitoring of renal function is essential during the usage of these agents [58].

Like valaciclovir, famciclovir is an orally administered prodrug that converts to penciclovir in the liver. Penciclovir, through competitive inhibition of the viral DNA polymerase, obstructs viral DNA synthesis [59].

At concentrations that do not impact human DNA polymerases, foscarnet selectively hinders the pyrophosphate binding sites on viral DNA polymerases. It is dissimilar from aciclovir and valaciclovir since it does not require viral kinases for activation, which renders it effective for aciclovir-resistant HSV strains [60]. Foscarnet is available for intravenous or intravitreal administration, however, its usage may lead to side effects such as nephrotoxicity, and neurotoxicity, and may also increase the risk of endophthalmitis, vitreous haemorrhage or RD with intravitreal administration [37].

Ganciclovir is an antiviral drug that restricts the activity of viral DNA polymerase. It can be administered intravenously, orally (in the form of valganciclovir), or intravitreally. However, the usage of ganciclovir is limited due to the need for a compounding pharmacy to prepare the medication [37]. Furthermore, systemic use of ganciclovir and valganciclovir may lead to severe side effects such as bone marrow suppression and secondary cytopenias [58].

#### Intravitreal antiviral treatment

Combining systemic and intravitreal antiviral therapy has been shown to effectively improve visual acuity and limit the progression of retinitis in ARN [30]. It also reduces the risk of retinal detachment. Although intravitreal antivirals like ganciclovir or foscarnet provide immediate and direct treatment for active infections, they cannot be used as monotherapy without systemic treatment due to the risk of contralateral involvement. Increasing the number and duration of intravitreal injections until the viral load in the aqueous humour is undetectable has been linked to an improved prognosis, such as reduced risk of retinal detachment and improved visual acuity [61]. Intravitreal foscarnet (2.4 mg in 0.1 mL) is increasingly being used in conjunction with oral antiviral agents, according to various case series [62, 63]. The immediate attainment of therapeutic vitreous drug levels and the inhibition of viral replication are the key benefits of intravitreal therapy. Moreover, foscarnet’s efficacy against herpes strains that are resistant to other drugs provides an additional advantage [42]. In refractory cases, therapy escalation should be considered, and several case reports have shown that patients responded well to intravenous foscarnet after initial treatment failure [64]. Furthermore, intravitreal ganciclovir has been found to have a synergistic effect with foscarnet, improving outcomes in recalcitrant cases [65].

#### Topical and systemic corticosteroids

Since ARN typically induces a severe inflammatory response, the use of systemic and topical steroids can be considered in conjunction with antiviral therapy. However, administering immunomodulatory agents to patients with severe inflammation requires caution since it may exacerbate viral replication and consequently hasten retinitis progression if initiated too soon [66]. Typically, patients are started on topical steroids during the initial treatment phase, while oral steroids may be added to the treatment regimen 24–48 h after antiviral therapy has been initiated. The most common approach involves a loading dose of 0.5 mg/kg/day of prednisone [58]. Nonetheless, there is presently no conclusive proof to substantiate this treatment regimen and no research has contrasted the consequences of eyes treated solely with antiviral drugs to those treated with a combination of oral corticosteroids and antiviral medication. In the event of use, oral corticosteroids should always be accompanied by antiviral medication since they may foster viral replication. It has been proposed that the use of oral corticosteroids can help minimize vitritis and lower the risk of RD [67]. Certain case reports have suggested the use of intravitreal triamcinolone after the onset of antiviral therapy [68]. However, no comparative studies have been released to address this issue. Although dexamethasone implants have also been shown to be effective in controlling intraocular inflammation and treating its complications (such as cystoid macular oedema) [65] pose several risks and are not generally recommended.

#### Prophylactic laser photocoagulation, vitrectomy, and other surgical considerations

Due to the high incidence of retinal detachment (RD) in cases of acute retinal necrosis (ARN), even with appropriate treatment [69], preventive measures such as laser photocoagulation and pars plan vitrectomy (PPV) have been implemented to minimize the risk of secondary RD and retinal tears.

The use of prophylactic laser retinopexy has been suggested as a means of reducing this risk [11, 12, 70]. However, the literature on this matter is debatable, as several studies have reported little to no benefit for patients with the use of prophylactic laser [21, 71]. A prophylactic laser barricade can create strong adhesions between the chorioretinal tissue and the affected retinal areas, and some studies have shown a statistically significant decrease in the incidence of RD in eyes that have undergone prophylactic laser therapy. Chen et al. [72] conducted a systematic review and meta-analysis to assess the efficacy of laser photocoagulation in preventing RD in ARN. Their findings suggest that prophylactic laser barricade, when used in combination with antiviral agents and steroids, can be a valuable therapeutic tool. However, the interpretation of these results may be affected by selection bias, as patients who receive laser treatment may have less severe disease with lesions that are more responsive to photocoagulation [11, 12].

The available studies suggest that early PPV has variable outcomes, and its role in preventing RD or improving VA has yet to be fully elucidated. Risseeuw et al. [71] conducted a retrospective study to assess the effectiveness of prophylactic laser or PPV in reducing the risk of RD in ARN patients. The study included 63 ARN cases, and the results indicated that the rate of RD was higher in those who received preventive laser (45.5%), lower in those who did not receive any preventive treatment (26.7%), and lowest in patients who underwent prophylactic PPV (14.3%). Therefore, the utilization of prophylactic laser retinopexy to prevent RD in patients with ARN is still not widely adopted. PPV, which can eliminate inflammatory mediators and vitreous traction, is commonly combined with silicone oil tamponade to prevent RD. However, the efficacy of early PPV varies across studies due to differences in patient characteristics and follow-up periods [71]. Although one study demonstrated a reduction in RD with PPV, it did not lead to improved final VA compared to patients who did not undergo PPV [26]. In contrast, Luo et al. [73] found both a decrease in RD and an improvement in final visual acuity in the PPV group. Other studies by Ishida et al. [74] and Liu et al. [75] did not find a significant difference in recurrent RD or final visual acuity.

Regarding the management of RD secondary to ARN, Wu et al. [76] conducted a recent study that suggested that modern vitreoretinal surgical techniques may result in moderate anatomic success in a single surgery for ARN-related RD; however, visual outcomes tend to be unsatisfactory. Therefore, it is necessary to perform a detailed preoperative assessment to determine the activity of the inflammatory process, which may impact the dosing of intravitreal antivirals, particularly in silicone oil-filled eyes. Based on our clinical and surgical experience, surgical interventions should be delayed until inflammation has subsided. If the RD is localized and not affecting the macula, surgical repair of the RD can be performed concurrently with an intravitreal antiviral injection at the time of silicone oil instillation. The surgical approach comprises PPV, endolaser, and silicone oil tamponade, which is generally preferred over long-acting gas therapy due to the higher risk of complications such as recurrent RD and the development of PVR. According to a recent meta-analysis, prophylactic vitrectomy may decrease the incidence of RRD, but the use of silicone oil tamponade and the risk of long-term complications may have negative effects on the patient’s visual outcome [77]. In certain cases, scleral buckling may be a viable option, but a number of factors must be considered (e.g., the severity of retinitis at the posterior pole, the extent of RD in clock hours) before determining whether a scleral buckle would be advantageous [2].

#### Long-term prophylaxis with antiviral therapy

The role and duration of long-term prophylactic antiviral therapy in ARN patients have not yet been defined precisely. After administering 2 g of Valaciclovir three times daily for 2 weeks, followed by 1 g of valaciclovir three times daily for 3 weeks, then a long-term prophylactic treatment can be offered to the patient. Some experts have suggested using oral valaciclovir for an extended period, even lifelong, to prevent the fellow eye’s involvement. While there is limited evidence supporting the efficacy of long-term antiviral therapy for herpetic retinitis, one study found that IV aciclovir decreased the involvement of the unaffected eye from 70% to 13% [5]. Our analysis in patients with uveitis showed that a 1-year treatment period with oral aciclovir reduced the recurrence rates of herpetic ophthalmic disease. However, extending the treatment for more than 1 year did not provide significant benefits [44]. The appropriate dosage for long-term valaciclovir use ranges from 500 mg to 1 gram twice per day, depending on several factors such as the patient’s visual acuity in the affected eye, the status of the functional eye, kidney function, and other herpes-related comorbidities (e.g., history of genital herpes, HSV encephalitis, or oral ulcers) [78]. These decisions are made on a case-by-case basis, considering the patient’s overall health, the medication’s potential risk, and the unaffected eye’s potential risk.

#### Other therapeutic considerations

Retinal ischaemia caused by retinal vascular occlusions is common in ARN-affected eyes. Adenosine 5-diphosphate aggregation testing and partial prothrombin times have revealed hyperaggregation of platelets in seven patients with bilateral ARN [79]. While the use of anticoagulants such as heparin and warfarin lacks sufficient evidence, platelet hyperaggregation can be treated with aspirin and steroids [79]. However, it is always crucial to carefully assess the patient’s overall health status and physical condition before considering the use of these medications.

Furthermore, the inflammatory environment may cause the inner and outer blood-retinal barrier to break down, which can lead to cystoid macular oedema (CMO). Currently, there is no established treatment for CMO in ARN patients. Bograd et al. [80] reported the combination of tocilizumab and intravitreal aflibercept for the treatment of refractory ARN-related CMO in a 14-year-old female. The use of aflibercept in the management of ARN-related macular oedema was also reported by Ortega-Evangelio et al. [81]. Their patient experienced consistent decreases in macular thickness and a 3-line improvement in VA after each injection.

Pegaptanib and interferon-α-2 are among the off-label therapies that have been found useful in treating CMO caused by ARN [82, 83]. However, they are not currently approved for ophthalmic use in many countries, aflibercept is the preferred treatment for CMO post-ARN. This medication is a soluble protein that blocks placental growth factor and all isoforms of vascular endothelial growth factor (VEGF) and has a longer half-life and a stronger affinity to VEGF-A than bevacizumab, pegaptanib, or ranibizumab [81].

### Patients’ age, immune status, and other comorbidities

ARN is primarily observed in adults; however, cases of ARN in children have also been documented [84]. Age is an important factor in predicting the visual prognosis, with older individuals (>80 years old) having a poorer prognosis compared to middle-aged patients [85]. The patient’s immune status should always be considered in ARN cases [86], especially in those who are immunosuppressed or immunocompromised (e.g., those undergoing biologic therapy), and a comprehensive ophthalmic assessment should be performed when visual loss is reported [87–89]. Although some studies have reported cases of ARN in patients with recent SARS-CoV-2 infection, further research is needed to establish a definite association [90]. Additionally, ARN has been linked to COVID-19 vaccination, as reported by other researchers [91]. Other potential causes of ARN reported in the literature include herpes zoster vaccination [92], cervical epidural steroid injection [93], intravitreal dexamethasone implant [94], cataract surgery [95], and intravitreal ranibizumab for exudative macular degeneration [96].

### Future perspectives

As underlined above, prompt identification and precise evaluation of the necrotic retinal area are critical for the diagnosis and treatment of ARN. Feng et al. [97] were the first to highlight the potential application of artificial intelligence (AI) algorithms in ARN. Their analysis created a computational algorithm for the automated detection and assessment of retinal necrosis from retinal fundus photographs. Subsequently, a novel algorithm based on deep machine learning was constructed for the detection and evaluation of retinal necrosis. The algorithm had an area under the receiver operating curve of 0.92, with 86% sensitivity and 88% specificity in detecting retinal necrosis. Regarding retinal necrosis evaluation, necrotic areas calculated by the AI algorithm were significantly and positively correlated with viral load in aqueous humour samples (R2 = 0.7444, *P* < 0.0001) and therapeutic response of ARN (R2 = 0.999, *P* < 0.0001). Therefore, utilizing retinal imaging and applying AI algorithms in these areas of clinical research could be highly beneficial.

Zhao et al. [98] assessed the effectiveness of xTAG liquid chip technology (xTAG-LCT) in identifying the causative agent of ARN. A total of 18 ARN patients provided 15 vitreous and 3 aqueous samples, which were analysed using both xTAG-LCT and multiplex PCR (mPCR)/quantitative PCR (qPCR). The xTAG-LCT detected a positive result in 17 out of 18 samples, revealing VZV as the sole cause in 10 samples, VZV and EBV in 5 samples, HSV-1 and EBV in 1 sample, and VZV, HSV-1 and EBV in 1 sample. In comparison, mPCR yielded the same results as xTAG-LCT for VZV and HSV-1 in all samples, but only 2 of the 7 samples that xTAG-LCT detected as positive for EBV were confirmed by qPCR. None of the 28 control vitreous samples from 8 non-ARN patients and 10 pairs of cadaveric eyes tested positive for any of the viruses. Therefore, xTAG-LCT may serve as a beneficial alternative for diagnosing the aetiology of ARN.

In recent times, the application of biologics as a treatment option for ARN has gained attention, even though it has not been widely adopted yet. Given the rising incidence of aciclovir (ACV)-resistant strains among patients with ocular HSV infections is a significant public health concern in developed nations. To address this issue, Bauer et al. [99] investigated the effectiveness of the humanized monoclonal antibody (mAb) hu2c, which targets the HSV-1/2 glycoprotein B, in treating ACV-resistant infections of the eye using a mouse model of acute retinal necrosis (ARN). In this study, BALB/c mice were infected with an ACV-resistant clinical isolate via microinjection into the anterior eye chamber to induce ARN and treated systemically with mAb hu2c either 24 h before infection (pre-exposure prophylaxis) or 24, 40, and 56 h after infection (post-exposure immunotherapy). Control mice that received no treatment and those treated with ACV exhibited significant retinal damage, whereas mice treated with mAb hu2c were almost entirely protected from developing ARN. Based on these findings, mAb hu2c may prove to be a viable therapeutic option for individuals with drug-resistant or ACV-resistant ocular HSV infections, potentially averting blindness.

## Conclusion

This review aims to discuss the recent advancements in the diagnosis and treatment of ARN, a rare viral uveitis syndrome that can result in significant visual impairment despite proper management. Patients must comply with treatment and undergo close follow-up to achieve optimal visual outcomes. Prompt initiation of systemic treatment is necessary for suspected ARN cases. PCR testing has greatly improved the ability to diagnose the disease promptly and accurately. Currently, systemic (intravenous or oral) antivirals with adjunctive intravitreal antiviral therapy are recommended as first-line therapy to reduce disease severity, the risk of vision loss, and retinal detachment incidence. While intravitreal agents can manage the disease locally, additional research is needed to refine the frequency and duration of antiviral therapies. The use of steroids as an adjunctive therapy can help reduce inflammatory activity, but the optimal dose and timing of systemic corticosteroid treatment remain unclear. Management of ARN necessitates a comprehensive understanding of the role of surgical repair of retinal detachment and patient counselling regarding the need for surgery and prognosis. Long-term maintenance of oral antiviral therapy is essential to prevent disease recurrence or contralateral involvement. Despite significant progress in diagnosing and treating ARN, further research is needed to improve visual outcomes in this challenging clinical condition.

## Summary

### What is known about this topic

- Acute retinal necrosis (ARN) is a rare but severe ocular condition primarily caused by herpesviruses, notably varicella-zoster virus and herpes simplex virus.
- Traditional management strategies for ARN include antiviral therapy with acyclovir or ganciclovir, along with systemic corticosteroids to mitigate inflammation.
- The diagnosis of ARN relies heavily on clinical manifestations such as peripheral necrotizing retinitis and vitritis, often supported by laboratory tests such as polymerase chain reaction (PCR) for viral DNA.
- Despite these efforts, ARN remains challenging to treat due to its propensity for rapid progression and potential for significant visual morbidity.

### What this study adds

- Update on the management of ARN. The role of newer antiviral agents (such as foscarnet), which have shown efficacy in cases resistant to conventional therapy.
- The role of flow cytometry in the diagnostic approach. Utilization of flow cytometry to analyze vitreous samples for cellular composition, aiding in the differentiation of infectious versus non-infectious causes of uveitis.
- Diagnostic and therapeutic tips.
- Summary of future perspectives.
